# Small-Angle Particle Counting Coupled Photometry for Real-Time Detection of Respirable Particle Size Segmentation Mass Concentration

**DOI:** 10.3390/s21175977

**Published:** 2021-09-06

**Authors:** Rongrui Zhang, Heng Zhao

**Affiliations:** Centre for Lidar Remote Sensing Research, School of Mechanical and Precision Instrument Engineering, Xi’an University of Technology, Xi’an 710048, China; 2190220122@stu.xaut.edu.cn

**Keywords:** small angle, light scattering, photometry, real-time measurement, particulate matter, mass concentration

## Abstract

Respirable particulate matter air pollution is positively associated with SARS-CoV-2 mortality. Real-time and accurate monitoring of particle concentration changes is the first step to prevent and control air pollution from inhalable particles. In this research, a new light scattering instrument has been developed to detect the mass concentration of inhalable particles. This instrument couples the forward small-angle single particle counting method with the lateral group particle photometry method in a single device. The mass concentration of four sizes of inhalable particles in the environment can be detected simultaneously in a large area in real-time without using a particle impactor. Different from the traditional light scattering instrument, this new optical instrument can detect darker particles with strong light absorption, and the measurement results mainly depend on the particle size and ignore the properties of the particles. Comparative experiments have shown that the instrument can detect particles with different properties by simply calibrating the environmental density parameters, and the measurement results have good stability and accuracy.

## 1. Introduction

Generally, in atmospheric and air quality research, “PM_X_” refers to particles with aerodynamic diameter less than or equal to x µm. PM_10_ and PM_2.5_, etc., are commonly used to judge the air particulate matter (PM) pollution situation [1,2,3,4]. These particles will be deposited in different areas of the human respiratory tract after being inhaled by the human body, causing a great impact on human health [5,6]. Some particles smaller than 5 μm can be directly inhaled by the human body and cause damage to the respiratory system [7]. Some studies have found that the number concentration of particles in some environments is positively correlated with the concentration of bioaerosol [8]. At the same time, some scholars pointed out that areas with severe basic pollution have more deaths from COVID-19 [9,10]. Xie et al. showed that air pollution such as PM_2.5_ and PM_10_ is positively correlated with the probability of death of individuals infected with COVID-19 [11]. All these promote the comprehensive low-cost and efficient detection of the mass concentration of fine particles [2].

According to the 2020 EPA certification list published by the American Environmental Monitoring Technical Information (ATMIC) [12], the main methods for detecting particle mass concentration are the manual reference method, the manual equivalent method, and the automatic equivalent method. The first two methods directly measure the particle mass on the filter membrane and then divide by the sampling volume to obtain the mass concentration. Therefore, these two methods are the most reliable, but they cannot be measured efficiently with a long measurement period. The most widely used is the automatic equivalent method, mainly including the tapered element oscillating microbalance (TEOM) method [13], the β-ray method [14], and the light scattering method [15,16]. The TEOM method and the β-ray method have good accuracy and sensitivity, but their operations are complicated and expensive, and they must be equipped with a size selection inlet to remove particles outside the size. Compared with other measurement methods, the light scattering method has many advantages, such as fast measurement speed, high precision, good repeatability, online, and real-time non-contact measurement. The measurement of the mass concentration of suspended particulate matter in the air based on the light scattering method is mainly divided into two categories: one is the photometry to detect group particles [17,18], the other is the optical particle counter to detect a single particle [19,20,21]. Generally, the daylighting angle for detecting particle mass concentration by the light scattering method is mostly 90°. In fact, at this daylighting angle, some darker aerosol with strong light absorption (e.g., soot, black carbon, dust) will scatter a tiny amount of scattered light, which is about ten times less than some highly transparent particles (e.g., glass) [22,23,24]. In the measurement, some relatively dark particles will be ignored, and only brighter particles will be considered, which will cause measurement deviation. In addition, it has a strong dependence on the calibration of instrument parameters in different environments when using the traditional daylighting angle to detect particle mass concentration [21]. Both the photometric method and the particle counting method are very limited by the properties of the particles (complex refractive index and density). Before measurement, more complex calibration experiments are needed to determine the parameters of the instrument. When the measurement environment is quite different from the calibration environment, the measurement results often lose the reference.

Photometers and particle counters are generally considered as two very different instruments. However, in fact, the two instruments both inherently and simultaneously receive the photometric signal of group particles and the pulse signal of a single particle. Only one of them is analyzed. To ensure that a single particle appears in the photosensitive region, the particle counter usually works at a low concentration. The light signal scattered by some air molecules and the stray light of the instrument itself also constitute the system noise, which directly restricts the detection ability of the particle counter. The photometer has a good linear response in the high concentration range, but it also makes the single particle pulse signal submerged in the photometric signal. Both the photometer and the optical particle counter (OPC) can quickly monitor the mass concentration of particles in their original suspended state. Because the photometer does not need to consider the error caused by particle overlap, it can work well in the environment of high concentration and its calibration of the instrument are relatively simple [25,26,27]. OPC can invert the particle size information well and can measure the mass concentration of particles of different sizes at the same time without being restricted by the impactor cutter [28,29]. The photometer mainly detects the photometric voltage signal of the particle group when it works alone, so the particle overlap error can be ignored to some extent. When the OPC works alone, it mainly detects the scattering pulse signal of a single particle and can obtain the particle size distribution in real time. The photometer itself has a higher photometric response for smaller particle size close to the wavelength [28]. The OPC has a good signal-to-noise ratio for the scattering pulse signal of larger particles. This makes them have a complementary detection particle size range. The combination of the two makes the detected scattering light signal more resolved. In addition, the particle size information obtained by OPC can not only further reflect the particle quality information, but also can more intuitively classify the pollutants and determine the degree of harm to human body. The combination of photometer and particle counter can complement the strengths and weaknesses of the two measurement methods. It can obtain the particle mass concentration in different particle size range at the same time without the restriction of impactor cutter and has a high measurement concentration range. In addition, by receiving the scattering light signal at a small angle, the scattering light of the darker particles can be well detected and the measurement error can be reduced.

In this research, a new light scattering instrument for detecting particle mass concentration has been developed, which couples forward small-angle single particle method with lateral-angle photometric method into one device. The stray light signals caused by the light source or air molecules are suppressed or eliminated by the opto-mechanical structure optimization design and the adaptive phase-cancellation filtering algorithm. This instrument can detect the mass concentration of particles at different particle sizes in real-time by particle size segmentation, in a wide concentration range without using a particle impactor. Moreover, it can reasonably measure the darker particles. At the same time, the measurement results mainly depend on the particle size information and are insensitive to the change of particle properties.

## 2. Detection Theory

### 2.1. Photometry Method

The photometric signal mainly depends on the size, complex refractive index, and shape of particles. The photometer uses the output electrical signal of a photodetector to obtain the particle mass concentration through a conversion factor [17]. The Photometric signal conversion factor (*K_PSC_*) refers to the mass concentration of the unit particle corresponding to the unit luminous flux [18], which is usually calibrated in a certain particulate matter environment in the early stage. When the detection environment is quite different from the calibration environment, it is often necessary to recalibrate the coefficient. The photometric response is sensitive to particle size. When the particle size is close to the wavelength of the light source, the photometric signal is the strongest. For particles outside the size (*D* > 5*λ*), the photometric response decreases sharply, so PM_1_ is selected as the target particle size for photometric detection.

**Figure 1:**
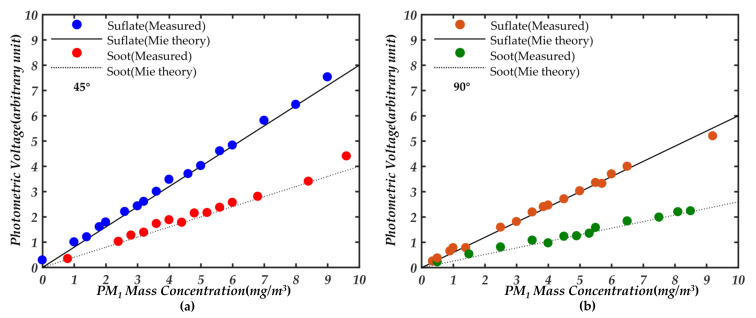
Linear relationship between aerosol mass concentration and the photometric response of soot and sulfate under actual measurement and theoretical calculation, respectively, (**a**) detection angle at 45°; (**b**) detection angle at 90°.

Mie theory can calculate the scattering luminous flux of a single spherical particle well. It is assumed that the minimum resolution of the developed instrument is 0.01 mg/m^3^ and the volume of the photosensitive area is *V_p_* = 1/6 × π*D*^3^, and *D* is the diameter of the spherical photosensitive area, we believe that the measured unit mass concentration is *C_m_*_0_ = 0.01 × *V_p_*. Without considering the multiple scattering of particles, it can be assumed that the detected unit group particles is a single particle of particle size *D_p_* = (0.06*V_p_*/π*ρ*)^−3^, and *ρ* is the particle density. Thus, using the Mie theory to obtain the scattering luminous flux *F*_0_ under the unit group of particles. The scattering luminous flux *F*_0_ of unit group particles can be converted into the scattering luminous power *P*_0_. By combining the scattering luminous power *P*_0_ per unit group of particles with the light sensitivity of the photodetector, the photocurrent *I*_0_ corresponding to the scattering luminous power of unit group particles is 0.6 A/W × *P*_0_. Combined with the magnification of the designed flow voltage circuit, the photometric voltage corresponding to the unit mass concentration of unit group particles can be obtained. That is, the slope of the photometric response under Mie theory is obtained. Then, we used standard instruments to detect the mass concentration of particles in the smoke box and recorded the photometric voltage at 45° and 90°, respectively, with the light detector. Thus, the photometric voltage corresponding to the particle mass concentration in the real environment is obtained. Based on the above analysis, the relationship between the photometric response and the mass concentration of soot and sulfate obtained by Mie theoretical simulation and actual measurement at 45° and 90° detection angles were obtained as shown in Figure 1. In the forward direction of 20° will receive a strong scattered light signal, which makes the dynamic range of the photometric change poor. From Figure 1, there is a good linear relationship between the photometric response of particles at the two receiving angles of 45° and 90° without considering the multiple scattering of particles. However, there is a good detection range at the 45° detection, so we choose the 45° angle to receive the photometric signal of the particles after careful consideration. Based on this linear relationship, it is simple to obtain the photometric signal conversion factor (*K_PSC_*) under different aerosol environments:

(1) $$K_{PSC}=\frac{C_{m,PM_{1},std}}{U_{P}}$$

where *C_m,PM_*_1*,std*_ represents the mass concentration of *PM*_1_ measured by the standard instrument, and *U_P_* represents the photometric voltage of the corresponding photodetector.

The *K_PSC_* obtained by calibration can be used to measure the mass concentration of *PM*_1_ by Equation (2):

(2) $$PM_{1}=U_{P}\times K_{PSC}$$

### 2.2. Optical Particle Counting Method

The instrument for detecting the mass concentration of particles based on the particle counting method is often an optical particle counter (OPC). When a single particle passes through the photosensitive region, the scattering light signal is generated by the electromagnetic wave action of the particle. At a specific angle, the photodetector receives the optical signal and converts it into an electrical signal. Due to the different sizes and properties of the particles passing through, the output electrical signals are a series of pulse signals [19]. The level of pulse signals can invert particle size information, and the number of pulses corresponds to the number of particles [20]. When OPC detects particles larger than 1 μm, the received pulse signals have a good signal-to-noise ratio. However, the pulse signals of some particles smaller than 1 μm are often submerged by noise signals. So, we use particle counting to detect particles larger than 1 μm.

#### 2.2.1. Forward Small Angle Detection

**Figure 2:**
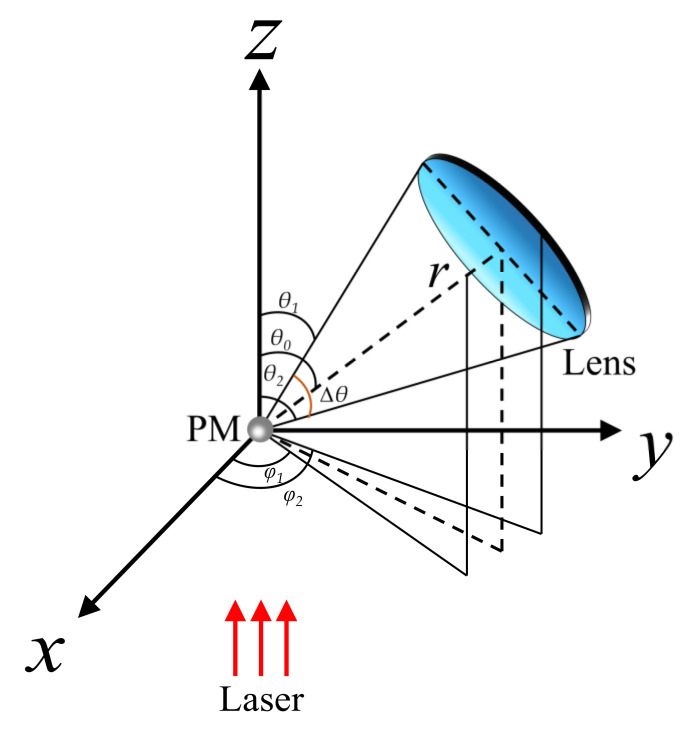
Luminous flux calculation for off-axis arrangement.

The relative luminous flux of particles refers to the scattering light energy collected by an inner lens at a solid angle per unit time. As shown in Figure 2, after a single spherical particle is irradiated by a linearly polarized light source in the photosensitive region, the scattering luminous flux *F* collected by the lens can be expressed as [30]:

(3) $$F=\int_{\varphi_{1}}^{\varphi_{2}}d\varphi \int_{\theta_{1}}^{\theta_{2}}I_{s}r^{2}\sin\theta d\theta$$

(4) $$F=\frac{\lambda^{2}I_{0}}{4\pi^{2}}\int_{\theta_{1}}^{\theta_{2}}\left[i_{1}(D,\lambda ,m,\theta )+i_{2}(D,\lambda ,m,\theta )\right]\Delta \varphi \sin\theta d\theta$$

(5) $$\Delta \varphi =\varphi_{2}-\varphi_{1}=\cos^{-1}\left[\frac{\cos(\frac{\theta_{2}-\theta_{1}}{2})-\cos(\frac{\theta_{2}+\theta_{1}}{2})\cos(\theta )}{\sin(\frac{\theta_{2}+\theta_{1}}{2})\sin(\theta )}\right]$$

where *I*_0_ is the light intensity of the laser, *I_S_* is the intensity of the scattering light at a point in space, *θ* is the scattering angle, Δ*θ* is the solid angle, *φ* is the azimuth angle, *r* is the distance from the particle to the lens, and *i*_1_, *i*_2_ are components of scattering intensity function which are functions of the scattering angle *θ*, the particle complex refractive index *m*, the particle diameter *D*, and the incident light wavelength *λ*.

In the Mie theory calculation, the incident wavelength is set to 650 nm and the particle shape is assumed to be spherical. Four particles with different properties and different light absorption were selected as: (1) sulfate particles with no imaginary part of refractive index (selected calcium sulfate with refractive index of 1.505); (2) glass beads with small imaginary part of refractive index (1.52 + 0.005*i*) [31]; (3) coal ash particles with a larger refractive index (1.50 + 0.012*i*) [32]; (4) and usually the deepest and most light-absorbing soot particles in atmospheric aerosols (2 + 0.6*i*) [33,34].

**Figure 3:**
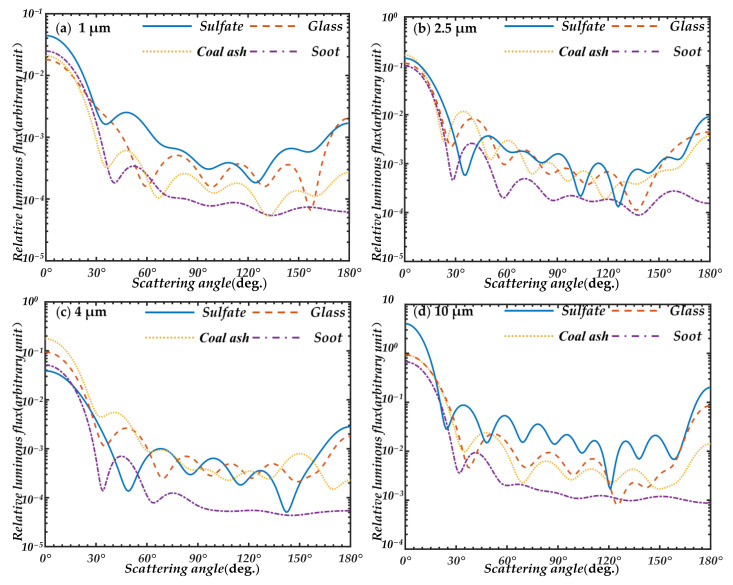
Results of Mie scattering calculations for four spherical particles of sulfate, glass, coal ash, and soot under different diameters, (**a**) 1 μm; (**b**) 2.5 μm; (**c**) 4 μm; (**d**) 10 μm.

In Figure 3, the scattering characteristics of these four particles at 1 μm, 2.5 μm, 4 μm, and 10 μm are calculated by Mie theory. To ensure that the distribution of particles is similar to that in the atmosphere, a relatively wide standard deviation is selected during calculation.

From Figure 3, the scattering characteristics of the particles with different absorbances are significantly different when the scattering angle is greater than 25°. In contrast, the scattering characteristics are similar and close to each other at small forward scattering angles. In the overall trend, the scattering characteristics of particles approach each other from 0°, roughly intersecting at about 15°. At about 30° they start to separate from each other again. Therefore, on the whole, the trend of particles with different properties and different absorption is similar at a small-angle with a scattering angle less than 30°, while the trend of particles with different properties fits tighter at about 15–25°. This indicates that the scattering characteristics of different particles in the small-angle range of 15–25° mainly depend on the size of particles and the differences caused by the particle properties can be ignored. The detection at a small angle can more accurately and solely determine the particle size.

**Figure 4:**
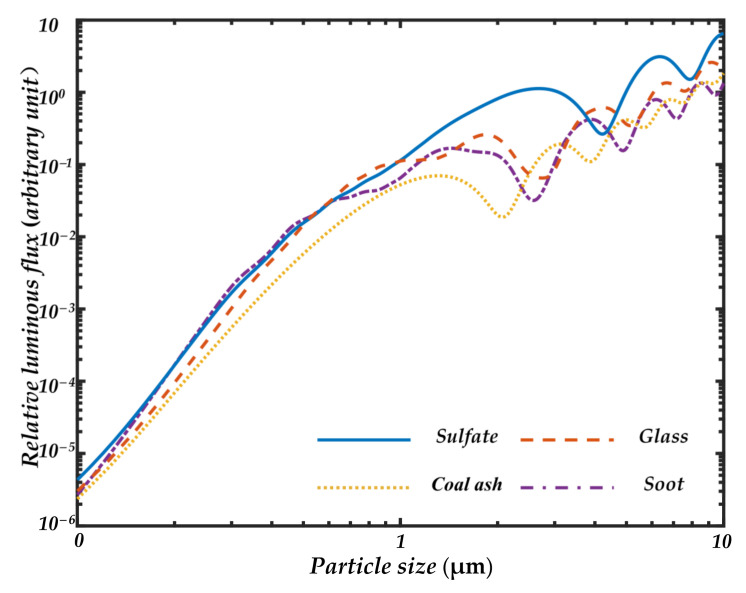
Mie scattering calculations for four spherical particles of sulfate, glass, coal ash, and soot at the scattering angle of 20 ± 5° in the range of 1 to 10 μm.

The forward measurement will be subject to a strong stray light caused by the light source, so a smaller solid angle of 10° has been selected for forward detection. Figure 4 shows the Mie scattering characteristics of the four kinds of particles varying in particle size from 0.1 to 10 μm at a detection angle of 20 ± 5°. Since standard spherical particles are used for simulation in the calculation, there are more oscillations on the curve [35]. The oscillations will be significantly reduced when some irregular particles are used in the real environment. As shown in Figure 4, the luminous fluxes of the four kinds of particles with different properties and different absorbances are very close to the trend of the particle size at 20°, indicating that the scattering luminous flux received at a small angle is mainly determined by the particle size. By receiving the forward 20° luminous scattering flux, the particle size values can be well inverted while ignoring the differences in particle properties.

#### 2.2.2. Inversion of Particle Mass Concentration by Single Particle Pulse

To use the forward single particle pulse to invert the particle mass concentration, it is necessary to assume that the light intensity distribution in the photosensitive region is uniform and the measured particles are all mean spherical particles. At the same time, the number concentration of particles in the measured range is diluted low enough by the sheath gas device, so that the overlap of particles can be ignored. When the particle size is continuously distributed in space, the spherical particle mass concentration *C_m_* can be written as the following integral form:

(6) $$C_{m}=\frac{\pi }{6}\rho \int_{D_{\min}}^{D_{\max}}L(D)D^{3}dD$$

where *ρ* is the density of spherical particles, *D* is the particle size, *D_min_* and *D_max_* are the smallest and largest particle size within the measured concentration, and *L*(*D*) is the particle size distribution, indicating that there are *L* particles of particle size *D*.

The particle size in the real environment is generally discontinuous. If the particle group with a finite total number of particles is considered, the mass concentration integral in Equation (6) needs to be converted into a summation form. In the actual measurement, the particle size measurement range [*D_min_*, *D_max_*] is equally divided into *n* sub-intervals, and the solution is calculated on each sub-interval. The larger the value of *n*, the smaller the difference between the integral formula and the summation formula. We use 2048 counting channels with equal voltage intervals to collect the pulse signals of the particles. Taking the height of the midpoint of each interval as the summed average height. Equation (6) can be approximated as:

(7) $$C_{m}=\frac{\pi }{6}\rho \sum_{i=1}^{n}L(D_{i}){D_{i}}^{3}\Delta D$$

In Equation (7), Δ*D* = (*D_max_* − *D_min_*)/*n*, *L*(*D_i_*)Δ*D* can represent the total number of particles in the range of *D_i_* − Δ*D*/2 to *D_i_* + Δ*D*/2 in a unit volume of air. Such that *N*(*D_i_*) = *L*(*D_i_*)Δ*D*, then:

(8) $$C_{m}=\frac{\pi }{6}\rho \sum_{i=1}^{n}N(D_{i}){D_{i}}^{3}$$

Mie scattering theory is a rigorous solution of Maxwell boundary conditions for particles under the action of electromagnetic waves [36]. However, in engineering applications, the approximate theory of Mie theory is mostly adopted in order to simplify calculations [37,38,39]. This not only makes the calculation simple without considering the complex recurrence relationship and the influence of the calculation order, but can also ignore the impact of variability caused by the oscillation of particle scattering characteristics. Reasonable use of the simplified model can greatly increase the computational efficiency. The detection range of our newly developed instrument is mainly 1–10 μm. In this particle size detection range, Fraunhofer diffraction theory can be used to simplify the relationship between *F* and *D*. Fraunhofer diffraction can be regarded as the approximation of Mie scattering theory for particle size in a large range. Based on Fraunhofer diffraction, the scattering light intensity *I_s_*(*θ*) at an observation point *P* of the receiving lens can be expressed as [38]:

(9) $$I_{S}(\theta )=\frac{I_{0}\lambda^{2}}{4\pi^{2}r^{2}}x^{2}{\left[\frac{J_{1}(x\theta )}{\theta }\right]}^{2}$$

where *x* = *πD*/*λ* is a dimensionless parameter, and *J_1_*(*xθ*) is the first-order Bessel function of *xθ*.

In combination with Equation (3), the scattering luminous flux *F* of the particles based on Fraunhofer diffraction is approximately proportional to the *D*^2^ [39]:

(10) $$F\propto D^{2}$$

In this particle size range, *F* and *D*^2^ can maintain a good linear and single value relationship. In practical application, we can estimate the approximate linear relationship between *F* and *D*^2^ according to the calibration experiment. Based on this approximate linear relationship, we can ideally use the received scattering luminous flux to retrieve the particle size. Generally, the output electrical signal *v* of the detector receiving the scattering light from a single particle is converted from a certain linear relationship between the scattering luminous flux *F* of the particle, and it is approximate as follows:

(11) $$v=kD^{2}$$

Substitute Equation (11) into Equation (8):

(12) $$C_{m}=\frac{\pi \rho }{6k^{1.5}}\sum_{i=1}^{n}N(v_{i}){v_{i}}^{1.5}$$

Integrating the constant term in Equation (12) as *K*, the particle mass concentration is:

(13) $$C_{m}=K\sum_{i=1}^{n}N(v_{i}){v_{i}}^{1.5}$$

where *v_i_* represents the pulse voltage in channel *i*, *N*(*v_i_*) is the number of *v_i_* voltages, and *K* is the coefficient that needs to be calibrated.

#### 2.2.3. Small Angle Particle Counting Coupled with Photometry

**Figure 5:**
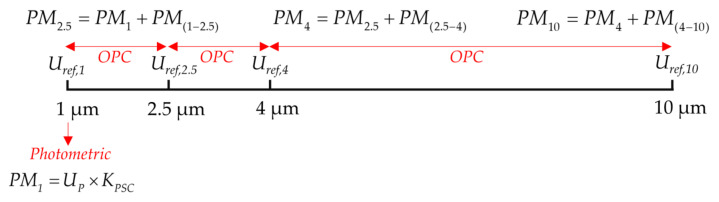
Forward small angle single particle counting coupled with photometry to detect the mass concentration of size segmented particles.

The limited voltage range collected by the photodetector is divided into 2048 voltage channels. Single-size particles of 1 μm, 2.5 μm, 4 μm, and 10 μm are passed into the photosensitive region at the air inlet, and the frequency of voltage occurrences in each channel is counted. The voltage channel with the highest probability of occurrence is determined as the reference voltage of the corresponding particle size, which is recorded as *U_ref,_*_1_, *U_ref,_*_2.5_, *U_ref,_*_4_, and *U_ref,_*_10_. As shown in Figure 5, 2048 equally spaced voltage channels are divided into three large particle size segments 1–2.5 μm, 2.5–4 μm, and 4–10 μm, according to the particle size range corresponding to each reference voltage interval. With the PM_1_ mass concentration measured by the photometric method, the mass concentration under each particle size separation can be obtained.

## 3. Instrument Design

### 3.1. Instrument Description

**Figure 6:**
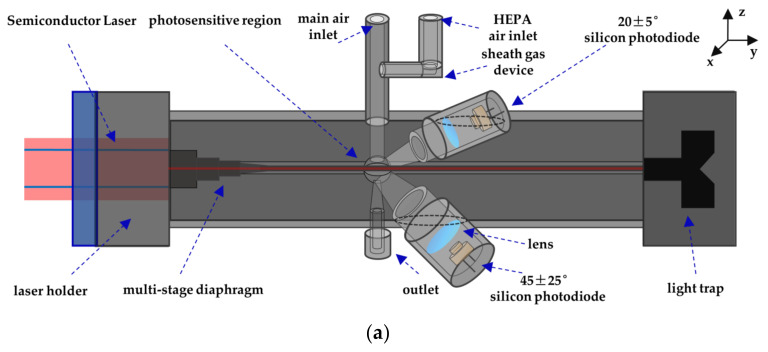

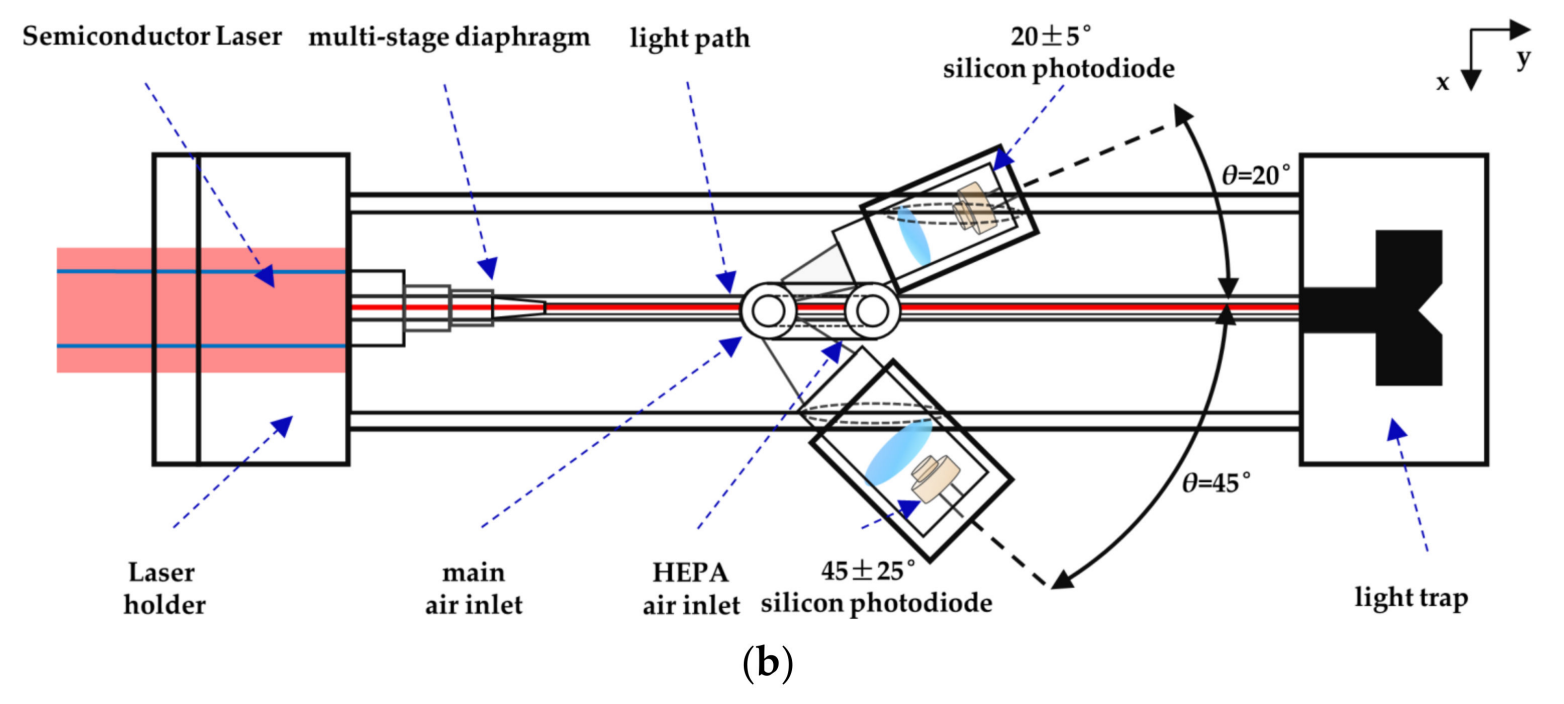
Schematic diagram of small angle single particle counting coupled photometry for detecting particle mass concentration prototype, (**a**) perspective view; (**b**) top view.

According to the measurement principle in Section 2, an engineering prototype of the forward small angle particle counter coupled photometric measurement system was designed. The schematic diagram of the prototype is shown in Figure 6. The gas path is divided into two parts. One part enters the sheath gas device and goes through high efficiency particulate air (HEPA) to remove particles. The number of particles is diluted about ten times by the sheath gas system. The rest part enters the photosensitive area after being neutralized by clean air. The light source selects a semiconductor laser with a wavelength of 650 nm and a power of 100 mw. The outgoing beam is focused by the lens group and changed from an ellipse to a rectangle through a multi-stage diaphragm. According to the selected wavelength of the semiconductor laser, the photodiode adopts S2386-44K (Hamamatsu Inc., Hamamatsu, Japan). After the particles in the photosensitive area are incident by the parallel laser beam, the scattered light is received by the forward 20° silicon photodiode and the lateral 45° silicon photodiode. The single particle pulse signal is received at 20° and is equipped with a small solid angle (10°) due to the larger light intensity received from the near forward angle. Because the dynamic range of the forward small angle photometric signal is small, the lateral angle of 45 ± 25° is selected to receive the group particle photometric signal.

**Figure 7:**
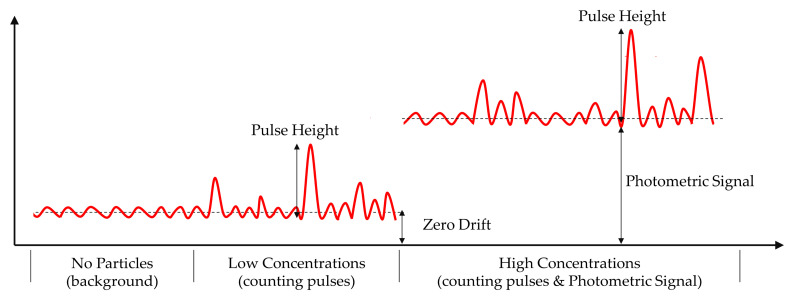
Pulse signal and photometric signal of particles at different concentrations.

The signal from the photodetector is processed by the Data Acquisition Card (ART Inc., Beijing, China) through the voltage bias analysis module and the single pulse height analysis module designed in LabVIEW. The photometric signal is received at 45° for estimating the mass concentration of the 1 μm particles, and the single particle pulse signal is received at 20° for estimating the mass concentration of the particle size range larger than 1 μm. Then the results of both calculations are integrated to obtain the particle mass concentration under each particle size segment. Figure 7 illustrates the signal of scattered light. The background noise of photodetector is mainly caused by the stray light due to the direct laser light source, the scattered light from air molecules, and the electrical noise of the system. At lower concentrations, the photometric voltage signal is almost zero, while at higher concentrations, a more stable photometric signal can be obtained. Pulse signals larger than the detection limit are recorded in height and times, and then inverted to obtain the information of particle size and quantity.

### 3.2. Suppression of Stray Light Background Noise

The lateral 45° is selected for detecting the photometric signal, which has good linearity and dynamic range. The forward 20° receiving the pulse signal of particles can not only detect the particles with strong absorbance, but also the measurement results mainly depend on the particle size. Compared with the traditional 90° receiving angle, the technique of forward small angle combined with lateral detection angle will be more severely affected by stray light and background noise. For such a forward small scattering angle of 20°, it is seriously disturbed by stray light. For this reason, we first chose to use nylon material with good light absorption as the material for 3D printing when manufacturing the light scattering shell and added black matte paint on the inner and outer surface to increase its absorption. In terms of optical path design, we use a multi-stage diaphragm to shape the beam to reduce halo and suppress reflected light. In addition, we designed an efficient light trap, which can suppress 90% of the stray light on the surface of the cavity wall by calculating the overall optical-mechanical structure.

**Figure 8:**
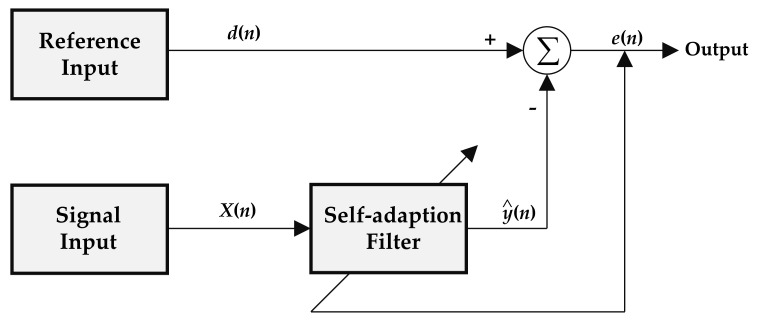
Principle block diagram of adaptive offset cancellation and suppression of forwarding stray light and background noise.

More importantly, an adaptive filtering algorithm has been developed to counteract forward stray light and to extract the light scattering signal of particles. The basic principle is shown in Figure 8. The reference signal without particle scattering after HEPA filtering and the detection signal with particle scattering during ventilation are taken as the two inputs of the filter. By adjusting the filtering parameters, the forward scattering noise in the air can be adjusted adaptively and offset each other. The pulse characteristics are highlighted in the received signal part of the detector, so that stray light and background noise are separated from the optical signal to obtain the scattered light signal. In the previous studies, the most commonly used adaptive cancellation algorithms are least mean square (LMS) and recursive least squares (RLS). The LMS algorithm is suitable for filtering and eliminating stationary random noise signals, but the tracking ability and convergence speed of the LMS algorithm for varying noise in a non-smooth environment is not as good as the RLS algorithm [40]. Considering that this stray light is affected by air molecules, reflected light in the cavity, and electrical noise, the RLS adaptively filter algorithm has been used to obtain the absolute value of pulse and photometric signal [41]. Due to space limitations in this paper, this section will not be the focus of our discussion.

### 3.3. Pulse Channel Division and Particle Size Segmentation

**Figure 9:**
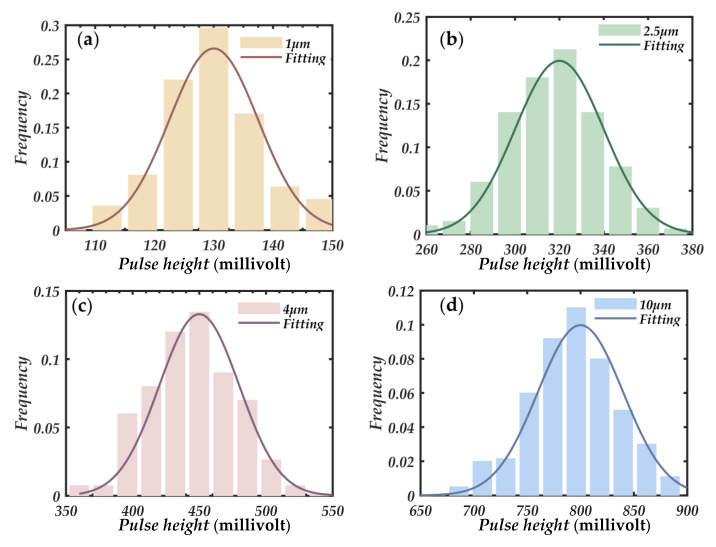
Four kinds of single distribution particle size pulse height frequency diagram, (**a**) 1 μm; (**b**) 2.5 μm; (**c**) 4 μm; (**d**) 10 μm.

In this measurement system, the photometer is used to detect particles with a size of 1 μm, and the particle counter is used to detect the mass concentration of particles in other particle size ranges. To obtain the mass concentration of particles for each particle size segment without using a particle impactor, determining the pulse reference voltage of each particle size segment is an important basis for dividing the particle size pulse channel. Four kinds of single-size particles of Thermo Scientific 4000 series 1 μm,2.5 μm, 4 μm, and 10 μm (Thermo Fisher Scientific Inc., Waltham, MA, USA) were used to calibrate the four pulse reference voltages of the measurement system. The selected standard size particles material was spherical polystyrene suspension solution, with the particle density of 1.05 g/cm^3^ and the refractive index of 1.59. Before use, dilute to about 1 × 10^3^/^mL^ with deionized water. The four kinds of sizes of particles were dispersed separately and evenly in the smoke box using an aerosol generator. The particles in the smoke box were sent into the photosensitive area through the vacuum pump. The Data Acquisition Card (ART Inc., Hong Kong, China) was used to count the pulse voltage and the corresponding number of times each particle appeared. From Figure 9, the four kinds of single-size particle pulses of different particle sizes are mainly divided into four voltage ranges. The frequency peak of each single particle size is taken as the reference voltage of corresponding particle size, noted as *U_ref,_*_1_, *U_ref,_*_2.5_, *U_ref,_*_4_, and *U_ref,_*_10_. According to the reference voltage range corresponding to the particle size range is divided into three large particle size segments 1–2.5 μm, 2.5–4 μm, 4–10 μm.

**Figure 10:**
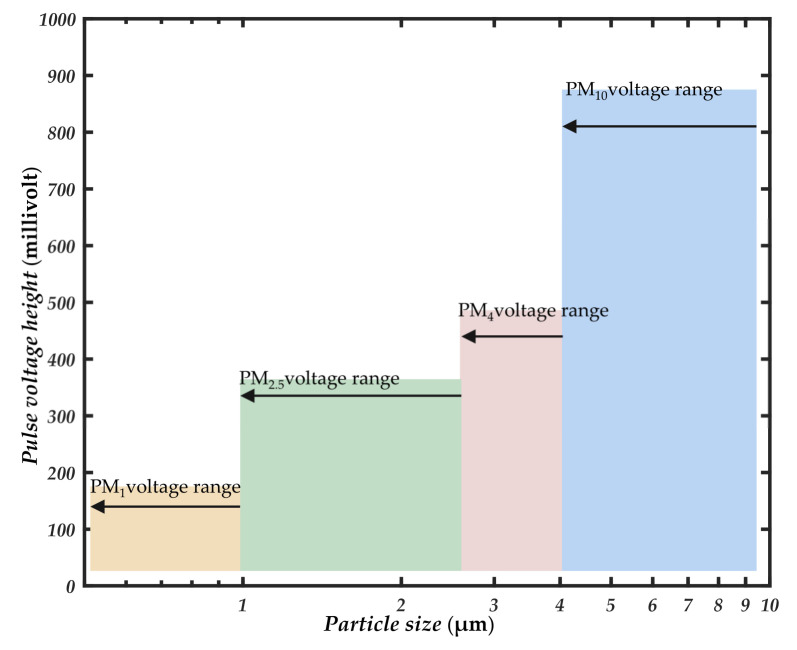
Particle size pulse reference voltage under different substances.

To verify the pulse reference voltage distribution of particles with different properties, another six particles with different properties were selected: fine sand, pulverized coal, lime, glycerin, DEHS, and glass. In the experiment, six kinds of particles were evenly dispersed in the smoke box using a polydisperse aerosol generator ARGE 8108 (TSI Inc., Shoreview, MN, USA). At the sampling port of the instrument, the aerodynamic standard impactor SCC cyclone cutter (BGI Inc., Bridgeview, IL, USA) was used to ensure again the photosensitive region of particles with a certain size range, and the scattering pulse voltage and frequency were recorded. In 2048 equal interval counting channels, only the highest pulse was reserved in each channel. As can be seen from Figure 10, there is no significant difference between the pulse voltage and the reference pulse height for particles with different properties in the same particle size range, that is, the scattering pulses of different particles in the same particle size range will not exceed the reference voltage threshold. This proves the validity of the reference pulse voltage of particle size calibrated by monodisperse standard dust and the applicability of particle measurement with different properties. At the same time, it shows that the forward small angle measurement can effectively ignore the influence of the complex refractive index of particles.

### 3.4. Calibration of Characteristic Parameters

In Equations (2) and (13), the constant parameters are integrated into a variable *K* and *K_PSC_*, respectively. Regardless of the particle counting method or the photometric method, the relationship between the electrical signal output by the photodetector and the required mass concentration is a certain nonlinear during actual measurement. Therefore, it is necessary to calibrate the characteristic parameters in the formula by nonlinear regression. For the nonlinear least squares method, an iterative algorithm is still a more effective regression method. A good iterative algorithm satisfies, fast convergence speed, low dependence on the initial value, small calculation amount, and wide applicability. Trust-region and Levenberg-Marquardt are typical algorithms for solving nonlinear optimization problems, which are used to improve the efficiency of inversion algorithm as much as possible and avoid falling into local optimum. Trust-region represents a subset of the objective function region approximated by the model function [42]. Its basic idea is the approximate solution of the quadratic subproblem whose increment is bounded by step size. Although the L-M algorithm cannot guarantee global convergence [43], it is more robust than the Gauss-Newton algorithm [44]. It can find the optimal solution even if its starting point is far from the final minimum. The least absolute residual (LAS) method was used to test the robustness of the parametric model.

Use the photodetector to obtain the *U_P_*, the scattering pulse voltage *v*, pulse number *N*(*v*), and the mass concentration value *C_m,std_* measured by the standard reference instrument. The standard reference instrument selected for calibration in the laboratory is DustTrack 8530 (TSI Inc., USA), a research-grade aerosol detector produced by TSI with a concentration range of 0.001–400 mg/m^3^. It is widely used in scientific research and industrial production. The experimental data were divided into seven groups according to the different calibration particles: fine sand, soot, lime, salt, glycerin, DEHS, and glass, each with 500 data points. Through the measurement of *U_P_*, *v*, *N*(*v*) and *C_m,std_*, *K*_0_, and *K_PSC_*_0_ are calculated according to Equations (14) and (15) as the initial values of trust-region and Levenberg-Marquardt algorithm.

(14) $$K_{0}=\frac{1}{T}\sum_{t=1}^{T}\frac{C_{m,std,t}}{\sum_{i=1}^{q}N_{t}(v_{i}){v_{i}}^{1.5}}$$

(15) $$K_{PSC0}=\frac{1}{T}\sum_{t=1}^{T}\frac{C_{m,std,PM1,t}}{U_{P}}$$

where *T* represents the number of groups of experimental data, and *t* represents the number of data points in each group of data.

As shown in Table 1, the initial values of seven groups under different particle size sections are obtained by Equations (14) and (15).

The *K*_0_ and *K_PSC0_* of different particle size segments in Table 1 were used as the initial values of the trust-region and Levenberg-Marquardt algorithms, and the upper and lower limits are set to inf and 0, respectively. The experimental data were fitted by nonlinear least squares. The results of the optimization of the model parameters were evaluated by the decidable coefficient R^2^ and the square sum of estimation (SSE). Optimization results are listed in Table 2. T-R and L-M algorithms obtain the same result, which indicates that the possibility of the optimization results falling into local optimum is reduced. From Table 2, the calibration coefficients are different under different material properties. However, for the forward small angle particle counting method, the numerical differences of each characteristic parameters are small under the same particle size segment. This again verifies that the light scattering signal of particles detected at a small angle in the forward direction can ignore the particle properties and mainly depends on the particle size. Meanwhile, it also proves the ability of the system to suppress stray light and the effectiveness of using an adaptive filtering algorithm to extract useful signals.

To effectively use the data of all calibration parameters and obtain the central tendency of the calibration data set, the calibration coefficients obtained under various particles are processed by arithmetic average. The processed parameters are shown in Table 3 and used as the calibration parameters of the measurement system.

## 4. Performance Test

**Figure 11:**
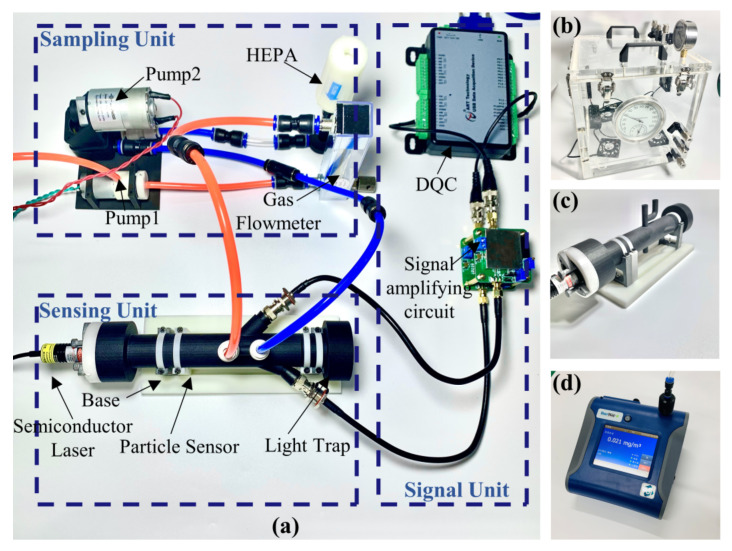
Small angle particle counting coupled photometric measurement system, (**a**) overall composition of the system; (**b**) smoke box; (**c**) detection prototype; (**d**) DustTrack 8530.

To test the performance of the forward small angle particle counting coupled with photometry in aerosol detection, a series of experiments were conducted, which includes a comparison with the standard light scattering instrument DustTrack 8530 through a simulated smoke box in the laboratory and a long-time comparison with the TEOM instrument TEOM 1405D in a real atmospheric environment. The overall experiment of the prototype is shown in Figure 11.

### 4.1. Comparison with DustTrack 8530 through the Simulated Smoke Box

To evaluate the pros and cons of the trust-region and Levenberg-Marquardt algorithms in calibrating the characteristic parameters, the parameters in Table 3 were substituted into the measurement system inversion model to detect the separation mass concentration of each particle size and compared with the standard instrument. In this experiment, six aerosols (sand, salt, glycerol, soot, glass, DEHS) were suspended in the smoke box by an aerosol generator. The aerosol concentration in the smoke box was changed by adjusting the airflow, and the smoke box was equipped with four fans to make the aerosol evenly dispersed. Our experimental prototype and DustTrack8530 were simultaneously sampled from the smoke box, and the electrical signal from the photodetector was amplified and sent into LabVIEW for processing. The sampling period, sampling frequency, and minimum signal amplitude can be set in LabVIEW, and the measurement period of the prototype was consistent with the DustTrack8530. The particle mass concentration measured by the prototype as *C_m,test_* was compared with the results *C_m,std_* measured by the standard reference instrument DustTrack8530, and linear regression analysis was conducted.

**Figure 12:**
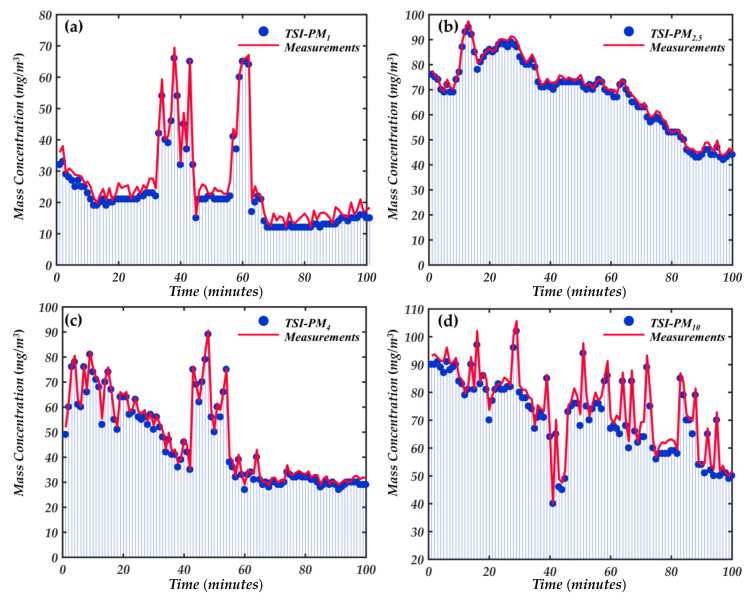
Real-time comparison of particle mass concentration changes in the smoke box between prototype and DustTrack8530 under different particle sizes, (**a**) PM_1_; (**b**) PM_2.5_; (**c**) PM_4_; (**d**) PM_10_.

**Figure 13:**
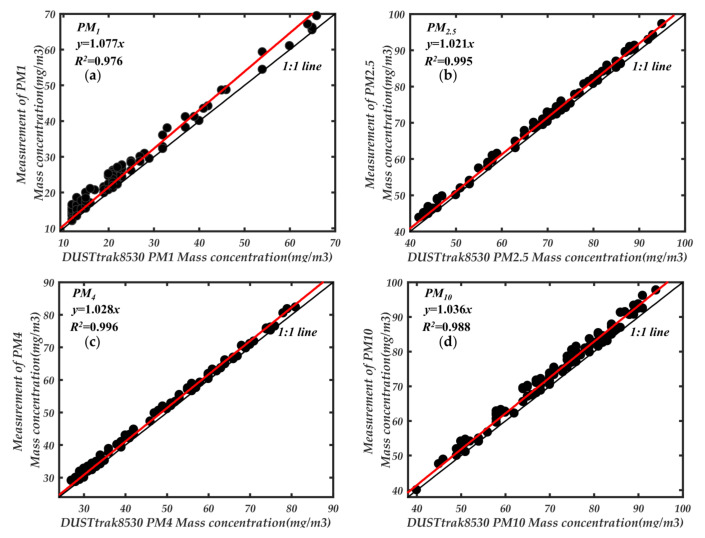
Linear regression between the prototype and DustTrack8530, (**a**) PM_1_; (**b**) PM_2.5_; (**c**) PM_4_; (**d**) PM_10_.

Figure 12 compares the mass concentration changes of mixed particles under different particle sizes, respectively. DustTrack8530 was equipped with an impactor in the air inlet corresponding to the particle size. From Figure 13, the four-particle size ranges mass concentration are in good agreement with the standard instrument 8530, and the consistency is about 8%. The results demonstrate the correctness of the trust-region and Levenberg-Marquardt algorithms for calibrating feature parameters.

**Figure 14:**
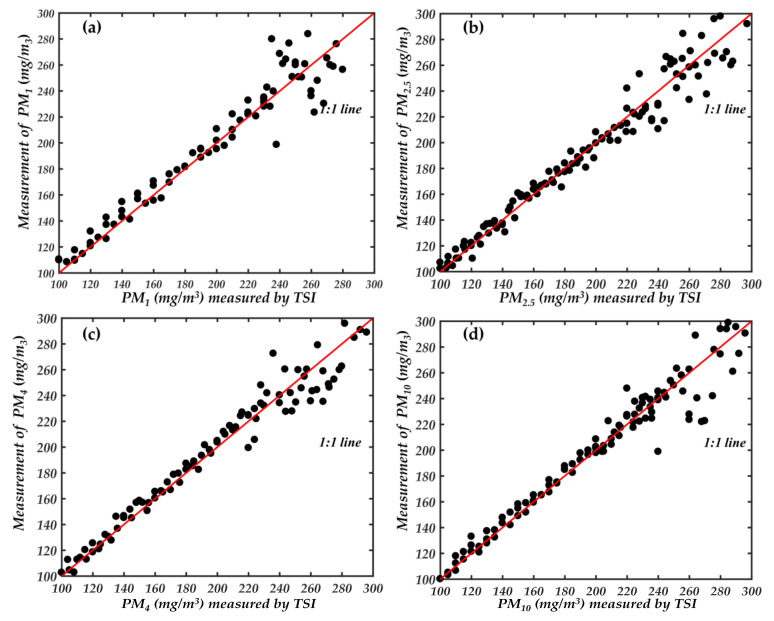
Comparison of detection mass concentration range, (**a**) PM_1_; (**b**) PM_2.5_; (**c**) PM_4_; (**d**) PM_10_.

In addition, Figure 13 reflects the detection range of our newly developed instrument to a certain extent. However, this is not the maximum detection range of the newly developed instrument in this paper. The Dusttrack 8530 is based on photometric detection of particle mass concentration, which has a wide concentration detection range of 400 mg/m^3^. We used it to compare the detection range with our newly developed instrument. From Figure 13, we can see that the newly developed instrument already has a good detection capability at particle concentration of 100 mg/m^3^. Therefore, we used the aerosol generator to control the particle concentration in the smoke box at about 100 mg/m^3^, and then started a comparison experiment with 8530 and gradually increased the particle concentration in the smoke box. The comparison results are shown in Figure 14.

From Figure 14, the points on the regression curve of different particle size ranges around 200 mg/m^3^ begin to shift. The different ranges of particles within 200 mg/m^3^ have good consistency. Therefore, the measuring range of newly developed instrument is about 200 mg/m^3^. Although the particle concentration range is lower than the traditional photometric method. However, compared with the traditional optical particle counting method of 1–10 mg/m^3^ has a significant improvement.

According to the derivation of Mie theory in Section 2 and the experimental verification of the smoke box, the designed forward small angle can well ignore the influence of the measurement results caused by the difference of particle complex refractive index. In the measurement of particle mass concentration, once particle size and complex refractive index are determined, the density becomes the key factor for accurate measurement. For this reason, an average density coefficient (*K_AD_*) is added when measuring in different environments. In the early stage, the measurement parameters calibrated by two non-linear least-squares methods through different aerosols are regarded as the inherent parameters of the measurement system. At this time, the *K_AD_* is equal to 1. Since the average density of particles in the environment is relatively stable at a given time, and density as a constant coefficient, the *K_AD_* can be obtained quickly by simple linear regression:

(16) $$K_{AD}=\frac{C_{m,std}}{C_{m,test}}$$

where *C_m,std_* represents the mass concentration measured by the standard instrument, and *C_m,test_* represents the mass concentration measured by the newly developed instrument.

### 4.2. Comparison with TEOM Instruments under Real Atmospheric Environment

To verify the instrument’s effectiveness for measuring particulate matter in a real external environment, it is necessary to conduct an external comparison test. The Xingqing District air quality monitoring station (108°54′58″ E, 34°17′3″ N) was selected as the standard reference point of the contrast test. The air quality automatic monitoring station of Xingqing District is hereinafter referred to as the monitoring station. For the detection of fine particles, it uses an EPA certified TEOM 1405D detector (Thermo Fisher Scientific Inc., USA) based on the TEOM method, which can effectively detect the mass concentrations of PM_2.5_ and PM_10_. The monitoring station is in the East Second Ring Road of Xi’an City, and most of the surrounding areas are residential buildings and businesses. The primary sources of pollutants are vehicle exhaust and human life emissions. Although the particle properties can be well ignored by using small angle detection, the density coefficient still needs to be calibrated when the environment is very different. Based on not changing the system’s parameters calibrated by various kinds of particles in the laboratory, the environmental average density coefficient was simply corrected by Equation (16) compared with the TEOM 1405D instrument. Based on the principle of light scattering to detect the mass concentration of particles, its accuracy is easily affected by the properties, shape, size, and density of particles. After calibrating the characteristic parameters, the mass concentration of particles can be measured stably under constant laboratory conditions. When detecting real atmospheric particles, the measurement results may have large deviations due to the variation of particle properties with time and geography. To ensure the accuracy of the measurement, we recalibrated the density coefficient twice a day at 8 a.m. and 8 p.m., respectively, during the long comparison observations. During the experiment, the inlet duct was used to ensure the same gas sampling between the prototype and TEOM instrument. To reduce the influence of relative humidity on the measurement results, the air inlet of the prototype was equipped with a drying tube to dry the gas during the measurement of atmospheric aerosol. The instrument was zero calibrated by HEPA filter before measurement, and the average zero drift of the measuring prototype was 1.6 ± 0.7 μg/m^3^ in 24 h. In the subsequent data processing, the zero-drift value was subtracted from the measured value of the prototype.

The atmospheric environment comparison experiment was observed for a total of fifteen days, from 1 April 2021, to 15 April 2021. The scattered light signals from two angles in the prototype were received by a photodetector and transmitted to LabVIEW for real-time processing through the data acquisition card. The prototype outputs a measurement result every 30 s. Considering the long-time continuous comparison observation, we averaged the 120 measurement results obtained every hour and recorded them. Similarly, the measurement cycle of TEOM 1405D was recorded once every hour.

**Figure 15:**
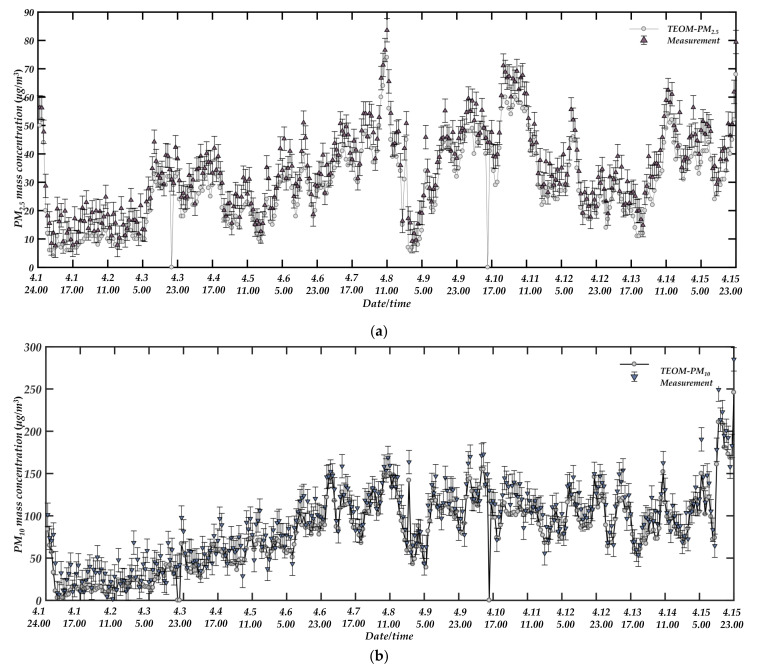
Continuous comparison observation between prototype and TEOM instrument in the real atmospheric environment, (**a**) PM_2.5_; (**b**) PM_10_.

**Figure 16:**
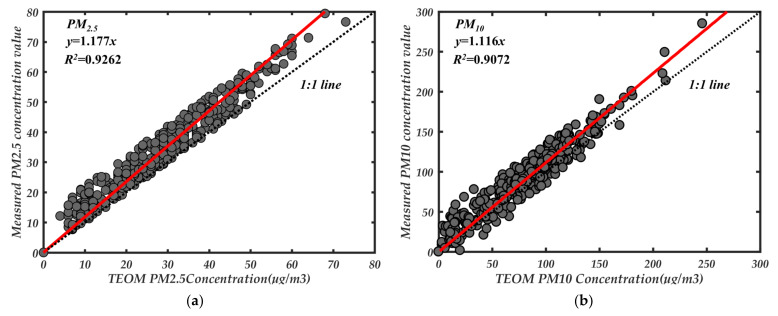
Linear fitting results of continuous observation between prototype and TEOM instrument, (**a**) PM_2.5_; (**b**) PM_10_.

Figure 15 shows the comparison of PM_2.5_ and PM_10_ measured by the prototype for fifteen consecutive days with the results measured by TEOM 1405D. Because the TEOM instrument was maintained at 20:00 on 3 April and 15:00 on 10 April and did not have measurement data, these points were deleted when doing regression analysis. Overall, the measured results have a good consistency. This is due to the use of forward small angle single particle technology combined with photometric method to reduce the impact caused by particle attributes, and due to the use of adaptive filtering to remove the impact of forwarding stray light and background noise in signal processing. When the dynamic change of particle properties in the external environment was different, it still maintained an excellent followability with the TEOM instrument. Figure 16 shows the linear fitting results corresponding to the hourly average mass concentrations of PM_2.5_ and PM_10_ continuously observed by the prototype. From Figure 16, the hourly average mass concentration measured by the prototype has a good correlation with the reference data of TEOM 1405D. Although it has better followability with the TEOM method, there is still a gap compared with the aerosol produced in the laboratory. Probably due to the poor sphericity and uniformity of the atmospheric particles in the field and the more complex types of particles.

## 5. Conclusions

A new type of light scattering instrument has been developed, which couples the small angle single particle counting method with the lateral photometry in one device to detect the mass concentration of four size-separated respirable particles in real-time. Lateral photometry is used to detect fine particulate matter of 1 μm, which is the most sensitive particle size range for the photometric response signal corresponding to the source’s wavelength. Small angle single particle counting is used to detect particles larger than 1 μm, when the single particle pulse signal has a good signal-to-noise ratio. The optimized optical-mechanical structure design combined with adaptive filtering can effectively eliminate the stray light caused by the forward light source and the scattered light of air molecules, and effectively extract the particle’s scattering pulse signal of small angle. To ensure the measurement accuracy and reduce the particle coincidence error, a HEPA sheath gas device was used to dilute the concentration of particles in the sampling photosensitive area by about ten times. This instrument has a wider detection range than optical particle counters and can provide more accurate particle size information than photometers. The measurement results of the forward small angle OPC can ignore the difference of particle properties well. At this point, the particle density becomes the key factor for accurate measurement of particle mass concentration. When the measured particle is very different from the mixed particle used in the laboratory calibration, recalibration is required to obtain the ambient average particle density coefficient. The experimental results show that the instrument can detect the mass concentration of real atmospheric particles with reasonable accuracy for a long time after the coefficients are calibrated regularly.

By comparing with the TEOM instrument under real atmospheric conditions, this instrument has good agreement with the TEOM measurement results. The TEOM is limited to only measuring the size range determined by the particle impactor, while this new instrument can simultaneously detect particle concentrations of four size separations without using the particle impactor and has a faster sampling speed and response time. Therefore, this new light scattering instrument can accurately, efficiently, real-time, and low-cost detect the mass concentration of inhalable particles.

## Figures and Tables

**Table 1:** Initial values of different particles in different particle size segments.

Aerosol	*K*_(1–2.5)0_	*K*_(2.5–4)0_	*K*_(4–10)0_	*K*_*PSC*0_	Lower	Upper
Sand	4.73 × 10^−3^	6.12 × 10^−4^	2.13 × 10^−4^	2.29 × 10^−3^	0	inf
Salt	4.81 × 10^−3^	8.11 × 10^−4^	2.51 × 10^−4^	6.54 × 10^−3^	0	inf
Lime	5.85 × 10^−3^	6.65 × 10^−4^	3.37 × 10^−4^	5.23 × 10^−3^	0	inf
Soot	6.42 × 10^−3^	8.05 × 10^−4^	5.78 × 10^−4^	13.72 × 10^−3^	0	inf
Glycerol	4.10 × 10^−3^	7.65 × 10^−4^	2.28 × 10^−4^	5.74 × 10^−3^	0	inf
DHES	5.28 × 10^−3^	7.44 × 10^−4^	3.77 × 10^−4^	5.99 × 10^−3^	0	inf
Glass	4.45 × 10^−3^	6.02 × 10^−4^	3.67 × 10^−4^	2.87 × 10^−3^	0	inf

**Table 2:** Results of parameter optimization of particles with different attributes using two nonlinear least squares methods.

Aerosol	Methods	*K*_1–2.5_	*K*_2.5–4_	*K*_4–10_	*K_PSC_*
sand	Trust-region	5.92 × 10^−3^	3.11 × 10^−3^	6.61 × 10^−4^	7.71 × 10^−3^
sand	Levenberg-Marquardt	5.92 × 10^−3^	3.11 × 10^−3^	6.61 × 10^−4^	7.71 × 10^−3^
salt	Trust-region	6.77 × 10^−3^	4.65 × 10^−3^	6.05 × 10^−4^	3.12 × 10^−3^
salt	Levenberg-Marquardt	6.77 × 10^−3^	4.65 × 10^−3^	6.05 × 10^−4^	3.12 × 10^−3^
Lime	Trust-region	6.51 × 10^−3^	3.64 × 10^−3^	6.59 × 10^−4^	5.94 × 10^−3^
Lime	Levenberg-Marquardt	6.51 × 10^−3^	3.64 × 10^−3^	6.59 × 10^−4^	5.94 × 10^−3^
Soot	Trust-region	7.58 × 10^−3^	5.32 × 10^−3^	7.87 × 10^−4^	9.24 × 10^−3^
Soot	Levenberg-Marquardt	7.58 × 10^−3^	5.32 × 10^−3^	7.87 × 10^−4^	9.24 × 10^−3^
Glycerol	Trust-region	5.35 × 10^−3^	3.92 × 10^−3^	5.61 × 10^−4^	4.64 × 10^−3^
Glycerol	Levenberg-Marquardt	5.35 × 10^−3^	3.92 × 10^−3^	5.61 × 10^−4^	4.64 × 10^−3^
DEHS	Trust-region	5.29 × 10^−3^	4.71 × 10^−3^	5.42 × 10^−4^	2.13 × 10^−3^
DEHS	Levenberg-Marquardt	5.29 × 10^−3^	4.71 × 10^−3^	5.42 × 10^−4^	2.13 × 10^−3^
Glass	Trust-region	4.23 × 10^−3^	3.24 × 10^−3^	6.13 × 10^−4^	3.08 × 10^−3^
Glass	Levenberg-Marquardt	4.23 × 10^−3^	3.24 × 10^−3^	6.13 × 10^−4^	3.08 × 10^−3^

**Table 3:** System calibration parameters after the weighted average.

Parameters	*K*_1–2.5_	*K*_2.5–4_	*K*_4–10_	*K_PSC_*
value	5.95 × 10^−3^	4.08 × 10^−3^	6.32 × 10^−4^	5.122 × 10^−3^

## Data Availability

Not applicable.
